# NOX4-Derived ROS Promotes Collagen I Deposition in Bronchial Smooth Muscle Cells by Activating Noncanonical p38MAPK/Akt-Mediated TGF-*β* Signaling

**DOI:** 10.1155/2021/6668971

**Published:** 2021-03-19

**Authors:** Binwei Hao, Ruiting Sun, Xiaotong Guo, Lili Zhang, Jieda Cui, Yumin Zhou, Wei Hong, Yanan Zhang, Jinxi He, Xiaoming Liu, Bing Li, Pixin Ran, Juan Chen

**Affiliations:** ^1^Department of Pulmonary and Critical Care Medicine, General Hospital of Ningxia Medical University, Yinchuan, Ningxia 750004, China; ^2^The State Key Laboratory of Respiratory Disease, National Clinical Research Center for Respiratory Diseases, Guangzhou Institute of Respiratory Disease, The First Affiliated Hospital, Guangzhou Medical University, Guangzhou, Guangdong 510120, China; ^3^Department of Pulmonary and Critical Care Medicine, Shanxi Bethune Hospital, Shanxi Academy of Medical Sciences, Taiyuan, Shanxi 030002, China; ^4^GMU-GIBH Joint School of Life Sciences, Guangzhou Medical University, Guangzhou, Guangdong 510120, China; ^5^Department of Thoracic Surgery, General Hospital of Ningxia Medical University, Yinchuan, Ningxia 750004, China; ^6^Department of Anatomy and Cell Biology, Carver College of Medicine, University of Iowa, Iowa City Iowa 52242, USA

## Abstract

**Background:**

Airway smooth muscle (ASM) remodeling is a hallmark in chronic obstructive pulmonary disease (COPD). NADPH oxidase 4- (NOX4-) mediated reactive oxygen species (ROS) production plays a crucial role in cell differentiation and extracellular matrix (ECM) synthesis in ASM remodeling. However, the precise mechanisms underpinning its pathogenic roles remain elusive.

**Methods:**

The expression of NOX4 and TGF-*β*_1_ in the airway of the lung was measured in COPD patients and the control group. Cigarette smoke- (CS-) induced emphysema mice were generated, and the alteration of *α*-SMA, NOX4, TGF-*β*_1_, and collagen I was accessed. The changes of the expression of ECM markers, NOX4, components of TGF-*β*/Smad, and MAPK/Akt signaling in human bronchial smooth muscle cells (HBSMCs) were ascertained for delineating mechanisms of NOX4-mediated ROS production on cell differentiation and remodeling in human ASM cells.

**Results:**

An increased abundance of NOX4 and TGF-*β*_1_ proteins in the epithelial cells and ASM of lung was observed in COPD patients compared with the control group. Additionally, an increased abundance expression of NOX4 and *α*-SMA was observed in the lungs of the CS-induced emphysema mouse model. TGF-*β*_1_ displayed abilities to increase the oxidative burden and collagen I production, along with enhanced phosphorylation of ERK, p38MAPK, and p-Akt473 in HBSMCs. These effects of TGF-*β*_1_ could be inhibited by the ROS scavenger N-acetylcysteine (NAC), siRNA-mediated knockdown of Smad3 and NOX4, and pharmacological inhibitors SB203580 (p38MAPK inhibitor) and LY294002 (Akt inhibitor).

**Conclusions:**

NOX4-mediated ROS production alters TGF-*β*_1_-induced cell differentiation and collagen I protein synthesis in HBSMCs in part through the p38MAPK/Akt signaling pathway in a Smad-dependent manner.

## 1. Introduction

Chronic obstructive pulmonary disease (COPD) is a major cause of chronic mortality, which accounts for over 3 million annual deaths worldwide [1, 2]. Based on previous large epidemiological studies, the number of COPD cases was 384 million in 2010, with a global prevalence of 11.75% [1, 2]. Despite substantial advances in the pathophysiological diagnostics and treatments of COPD, chronic airway inflammation, overwhelming oxidative stress, protease and antiprotease imbalance, cell apoptosis, and airway remodeling (ARM) have recently been suggested as main drivers in the pathogenesis of COPD [3, 4]. However, the mechanism underlying the pathogenesis of COPD is yet to be clarified.

Due to exposure to exogenous sources of reactive oxygen species (ROS), such as cigarette smoke (CS), air pollutants, or endogenously released ROS from leukocytes and macrophages involved in the inflammatory process, oxidative stress was increased in patients with COPD [5, 6]. ROS function as signaling molecules that play a major role in cell growth, differentiation, apoptosis, gene expression, and activation of cell signaling pathways [7, 8]. Excessive ROS accumulation can also induce oxidative stress and result in cell damage. Lungs are vulnerable to oxidative stress due to the relatively high-oxygen environment, increased blood supply, and exposure to environmental pathogens and toxins [9]. Several enzymes have been identified to be involved in the generation of ROS. Among these, the members of the nicotinamide adenine dinucleotide phosphate oxidase (NOX) family were regarded as the primary ROS-generating enzymes [8]. Although the accumulation of O^2-^ is the primary biological function of NOX, the signaling is mediated by H_2_O_2_. Notably, NOX4 is unique among NOX1–5 isoforms in generating H_2_O_2_. Strikingly, NOX4 is mainly expressed in smooth muscle cells, fibroblasts, and endothelial cells [10]; NOX4 exacerbates the progression of COPD mainly through oxidative stress [10]. Our previous studies demonstrated that abundant NOX4 protein was detected in ASM cells of small airways in COPD and was positively correlated with ASM cell proliferation and the deposition of the extracellular matrix (ECM), while it was inversely associated with pulmonary function [11]. However, the role of NOX4 in the pathogenesis of COPD is yet unclear.

The normal structure and function of the airway are important for respiratory and pulmonary physiology. The dysfunctional narrowing of the conducting airways and impaired relaxation occur in patients with COPD and asthma [12–16]. Recently, accumulating evidence demonstrated that the expression of NOX4 was significantly elevated in airway smooth muscle (ASM), resulting in enhanced ROS and oxidative stress in asthma patients and COPD subjects [10]. Together with our previous findings that NOX4 protein was elevated in ASM cells of small airways along with the disease severity in COPD patients [11], these studies implied that NOX4 plays a major role in ASM hyperplasia and hypertrophy in the lungs of chronic pulmonary diseases. However, the role of these molecules as a modulator of ASM hyperplasia and hypertrophy in oxidative stress is yet to be elucidated.

Accumulated evidence indicated that ECM protein is implicated in multiple signaling pathways. It acts as a bioactive entity that regulates many cellular activities able to affect the pathogenesis of lung diseases [17, 18]. For instance, ASM cells exhibited a proliferative phenotype in response to peptide mitogens when they were seeded on substrates of either fibronectin (FN) or collagen I (Col I) [19]. In a rat model of COPD, the stimulation of ECM components, such as collagen subtypes I and III, also displayed an ability to induce the proliferation, migration, and adhesion of ASM cells [20]. Therefore, we inferred that the changes in collagen I affect lung function and cell biology. Importantly, the dysregulation of collagen I may provide a positive feedback loop to drive airway remodeling. However, the underlying mechanisms also need to be investigated further.

TGF-*β* regulates multiple cellular processes, such as growth suppression of epithelial cells, alveolar epithelial cell differentiation, fibroblast activation, and ECM organization, which is tightly associated with tissue remodeling by enhancing oxidative stress in series airway disease [21–23]. Mechanistically, TGF-*β* is cleaved to form a mature TGF-*β* dimer noncovalently associated with latency-associated peptide (LAP). Then, the secreted complex is bound by latent TGF-*β* binding protein (LTBP), which can be stored in the extracellular milieu [24]. In a dimeric form, TGF-*β* binds to the type I receptor (TGF-*β*R-I, also known as ALK-5). TGF-*β*R-I effectuates by regulating the canonical Smad-dependent pathway. It phosphorylates the intracellular signal transducers, Smad2 and Smad3. Phosphorylated Smad2 and Smad3 interact with Smad4 and translocate into the nucleus. In addition to this canonical pathway, TGF-*β* also activates the mitogen-activated protein kinase cascades and RhoA-GTPase [25]. Previously, we found that the expression of TGF-*β* in both ASM cells and airway epithelial cells (AECs) was increased along with the disease severity in COPD patients, suggesting that the level of TGF-*β* protein in ASM of small airways was correlated with the expression of NOX4 [11]. However, the role of downstream molecules in oxidative stress for ASM remodeling is still uncertain.

In view of the functions of NOX4 in ROS production and ASM hyperplasia and hypertrophy in COPD airway remodeling, as well as the contribution of TGF-*β* in the disruption of oxidant/antioxidant balance in ASM cells, we hypothesized that TGF-*β* induces intracellular ROS production in ASM cells through a mechanism by upregulating NOX4, substantially leading to ASM hyperplasia and hypertrophy.

## 2. Materials and Methods

### 2.1. Ethics Statement

Human samples were collected with a protocol approved by the Ethics Committee for the Conduct of Human Research of Ningxia Medical University (NXMU-2015-205). Written consent was obtained from every individual according to the Ethics Committee for the Conduct of Human Research protocol. All participants signed informed consent for scientific research of clinical data during hospitalization and were provided written informed consent for the publication of the data. The Ethics Committee for the Conduct of Human Research at General Hospital of Ningxia Medical University approved the consent procedure for this study (NXMU-2015-205).

### 2.2. Subjects and Data Collection

A total of 28 patients with COPD and 21 of gender- and age-matched individuals with normal lung function were recruited in the General Hospital of Ningxia Medical University from March 2016 to September 2017. All enrolled individuals with or without COPD suffered from a suspected early stage of non-small-cell lung cancer (NSCLC) and undergone pneumonectomy or lobectomy. The diagnosis of COPD was essentially according to the criteria of the Global Initiative for Chronic Obstructive Lung Disease (GOLD 2015). The exclusion criteria were as follows: patients accompanied with (1) other chronic lung diseases, such as bronchial asthma, sleep apnea-hypopnea syndrome, bronchiectasis, pulmonary fibrosis, and interstitial lung disease, and (2) abnormal liver and kidney function, known ischemic heart disease, congestive heart failure, structural heart disease, prior thromboembolic disease, cerebrovascular disease, peripheral arterial disease, hepatitis, and autoimmune disease were excluded in this study.

Basic demographic information was collected using a specifically designed questionnaire after written informed consent was obtained. Standard pulmonary function testing was performed on all subjects before the surgical performance. The pulmonary function was ascertained by measuring the postbronchodilator forced vital capacity (FVC) and forced expiratory volume in one second (FEV1), using a MasterScreen PFT spirometer system (CareFusion, San Diego, CA, USA). The routine blood testing was performed before antibiotic treatment and automated differential counts including white blood cells, neutrophils, and lymphocytes. The neutrophil-lymphocyte ratio (NLR) was calculated and collected. The demographics and clinical data of individuals were collected.

### 2.3. Animal Experiments

In order to exclude the impact of sex hormones on the generation of the COPD mouse model, male mice were employed in this study. C57BL/6 mice (male, 6–8 weeks old, and 18–22 g) were purchased from the Animal Center of Guangzhou University of Chinese Medicine (Guangzhou, China) and housed in specific pathogen-free environment conditions under 12 h light/dark cycles with *ad libitum* access to standard food and water [26]. All experimental protocols were approved by the Ethics Committee of Animal Experiments of Guangzhou Medical University. Mice were exposed to cigarette smoke (CS) as described previously [27]. The control mice were exposed to clean air.

### 2.4. Histology

For human lung tissue, COPD and normal lung tissues were collected and the analysis of the small airway of histochemistry and immunohistochemistry was according to our previous description [11, 28]. Parameters of the ASM mass in the small airway were measured as an index of the area of ASM/transverse area of the small airway (WA%) as previously described [11, 28]. For the mouse model, the enlargement of alveolar spaces was quantified by the measure of the mean linear intercept (MLI) in the control and CS-exposed groups of mice in a blinded manner, as previously described [26]. The thickness of the small airway wall was analyzed according to methods described by our group previously. In brief, small airways cut transversely and, with a basement membrane perimeter less than 1000 mm, were examined. The indicator of airway remodeling was measured as an index of the small airway wall area/length (mm^2^/mm). At least five small airways were counted on each slide [26]. The histology was performed on 4.0 *μ*m thick paraffin sections of lung tissues of mice or human individuals. Sections were incubated with NOX4 (1 : 100), *α*-SMA (1 : 500), anti-TGF-*β*_1_ (1 : 250), and anti-collagen I antibodies (1 : 200) (see supplementary data for details (available here)).

### 2.5. Preparation of Cigarette Smoke Extract

CS extract (CSE) was prepared as described previously [27].

### 2.6. Cell Culture

Human bronchial smooth muscle cells (HBSMCs) were purchased from ScienCell Research Laboratories (Cat. No. 3400) (San Diego, CA, USA). After starvation for 24 h in DMEM/F-12 with 0.5% FBS, the cells were pretreated with PD98059 (10 *μ*M), SB203580 (10 *μ*M), LY294002 (10 *μ*M), and N-acetylcysteine (NAC; 1, 5, and 10 mM, respectively) for 1 h before the addition of TGF-*β*_1_ (2 ng/mL).

### 2.7. Western Blot Assay

Protein expression was ascertained by an immunoblotting assay using the Criterion Western Blot 132 system (Bio-Rad), and appropriate primary and secondary antibodies were utilized: rabbit polyclonal anti-NOX4 (1 : 500, Novus Biologicals, Littleton, CO, USA), rabbit polyclonal anti-*α*-SMA (1 : 1000, Abcam, Cambridge, UK), and rabbit polyclonal anti-collagen I (1 : 2000, Abcam). The relative expression level of proteins of interest was normalized to that of the GAPDH internal housekeeping control protein. Data are represented as fold change over the control.

### 2.8. ELISA

The content of TGF-*β*_1_ in HBSMC supernatants was measured by a commercial TGF-*β*_1_ human ELISA kit (R&D Systems) per the manufacturer's instructions. The absorbance was read at 450 nm on a microplate reader (Thermo Fisher Scientific, Finland).

### 2.9. Measurement of Intracellular ROS

Intracellular ROS levels were determined by measuring the mean fluorescence intensity of 2′,7′-dichlorodihydrofluorescein diacetate (H_2_DCFH-DA) per the manufacturer's manual (Cat. No. D399, Molecular Probes, USA).

### 2.10. Quantitative Real-Time PCR

Total RNA was extracted from cultured HBSMCs. Reverse transcription was performed using a PrimeScript RT Reagent Kit (Takara Bio, Kyoto, Japan). The expression of *NOX4*, *α*-*SMA*, and *GAPDH* mRNAs was determined by quantitative real-time PCR reaction using the SYBR Green PCR system (Takara Bio, Kyoto, Japan). The data were normalized to that of GAPDH and expressed as fold change over the control.

### 2.11. Immunofluorescence Staining

The cells were fixed with 4% paraformaldehyde for 15 min at room temperature, followed by the staining protocol (see supplementary data for details (available here)).

### 2.12. Small Interfering RNA (siRNA) Transfection

NOX4, Smad3, and sham control (NC) siRNA were obtained from GenePharma (Suzhou, China) and transfected into HBSMCs using Lipofectamine™ RNAiMAX (Thermo Fisher Scientific) for 6 h. Then, the cells were stimulated with 2 ng/mL TGF-*β*_1_ for the indicated time points.

### 2.13. Statistical Analysis

All data are presented as means ± standard deviations (SD) and analyzed using GraphPad Prism 7.0 software (GraphPad Software Inc., La Jolla, CA, USA). ANOVA with LSD was used for multiple comparisons. *P* value < 0.05 was considered statistically significant.

## 3. Results

### 3.1. Demographic Data

Twenty-eight patients with COPD were enrolled in this study, including 20 males and 8 females with a mean age of 59.68 ± 9.43 years. And twenty-one non-COPD patients were enrolled in this study, included 9 males and 12 females with a mean age of 55.43 ± 7.65 years. The WBC, neutrophils, and neutrophil-lymphocyte ratio (NLR) were significantly increased in the sera of COPD patients (6.72 ± 2.28, 4.20 ± 1.84, and 2.56 ± 1.22) compared to non-COPD patients (5.34 ± 1.08, 2.87 ± 0.71, and 1.72 ± 0.94), respectively (*P* < 0.01), but there was no difference in the lymphocytes between the COPD patients and the control. The value of parameters of pulmonary functions including FEV1, FEV1%pred, and FEV1/FVC% was significantly different in patients with COPD compared with non-COPD subjects (Table 1).

### 3.2. Elevated Expression of NOX4, *α*-SMA, and TGF-*β*_1_ in the Small Airways of COPD Patients

**Figure 1:**
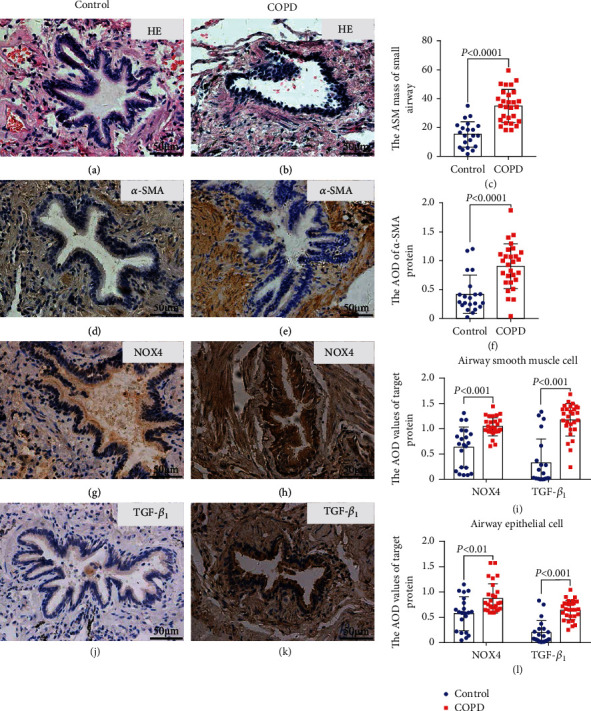
Elevated expression of NOX4, *α*-SMA, and TGF-*β*_1_ in the smooth muscle of the small airway of the human COPD lung. (a, b) Representative images depicted morphological changes as determined by HE staining of the lungs of the non-COPD lung (a) and COPD lung (b). (c) An increased area of airway smooth muscle (ASM)/transverse area of the small airway (WA%) in COPD patients (*n* = 28) compared to the non-COPD control small airway (*n* = 21). (d–f) Representative images of immunohistochemical staining (IHC) of *α*-SMA in the lungs of non-COPD patients (d) and COPD patients (e), and more abundant *α*-SMA was detected in COPD lungs over non-COPD lungs (f). (g–i) Representative images of immunohistochemical staining (IHC) of NOX4 in the lungs of non-COPD patients (g) and COPD patients (h), and more abundant NOX4 was detected in the airway smooth muscle cell (i) and airway epithelial cell (l) in COPD patients' lungs over non-COPD patients' lungs. (j–l) Representative images of immunohistochemical staining (IHC) of TGF-*β*_1_ in the lungs of non-COPD patients (j) and COPD patients (k), and more abundant TGF-*β*_1_ was observed in the airway smooth muscle cell (i) and airway epithelial cell (l) in COPD lungs relative to non-COPD lungs. Data was presented as mean ± SD.

Emerging research confirmed that the elevated expression of the TGF-*β*_1_ is evident in the airway smooth muscle of COPD patients [11, 29, 30]. Furthermore, TGF-*β*_1_ is a potent inducer of expression of NOX4, which triggers reactive oxygen species production, proliferation, and hypertrophy in cultured human airway smooth muscle cells [28]. We investigated the role of TGF and prooxidant enzyme NOX4 in COPD patients. The morphological analysis by HE staining revealed that an index of the area of airway smooth muscle (ASM)/transverse area of the small airway (WA%) in COPD patients was thicker than that in control patients (Figures 1(a)–1(c)). The expression of *α*-SMA (Figures 1(d)–1(f)) was increased in ASM cells of the lungs of COPD patients than the lungs of non-COPD individuals. More abundant NOX4 (Figures 1(g)–1(i)) and TGF-*β*_1_ (Figures 1(j)–1(l)) proteins in the epithelial cells and ASM cells were observed in the lungs of COPD patients than the lungs of control individuals by immunohistochemical staining (IHC).

### 3.3. Enhanced NOX4 and TGF-*β*_1_ Expression in Both Emphysematous Animal Models Induced by Cigarette Smoke (CS) and Cell Exposure to Cigarette Smoke Extract (CSE)

**Figure 2:**
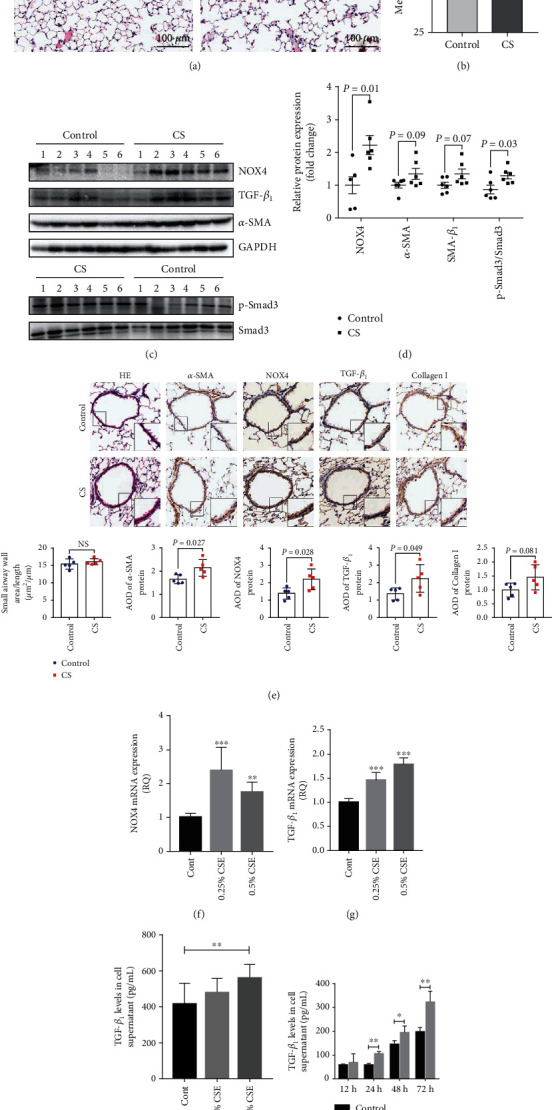
Elevated expression of NOX4 and TGF-*β*_1_ in CS-induced emphysema mice and CSE-treated HBSMCs. (a) Representative images depicted morphological changes as determined by HE staining of the lungs of mice exposed to clean air or CS. (b) The mean linear intercept of alveoli. (c) Protein expression of NOX4, TGF-*β*_1_, and *α*-SMA was measured by Western blot in the lungs of mice exposed to clean air (*n* = 6) or CS-induced mice (*n* = 6). (d) Relative to the expression of indicated proteins. (e) Representative images of HE staining and immunohistochemical staining of *α*-SMA, NOX4, TGF-*β*_1_, and collagen I in the lungs of mice exposed to clean air (*n* = 5) or CS-induced mice (*n* = 5). (f, g) The mRNA expression of NOX4 and TGF-*β*_1_ induced by indicated concentrations of CSE for 24 h. (f) NOX4 mRNA expression. (g) TGF-*β*_1_ mRNA expression. (h, i) The protein content of TGF-*β*_1_ induced by various concentrations of CSE for the indicated time points. (h) The levels of TGF-*β*_1_ induced by different concentrations of CSE. (i) The levels of TGF-*β*_1_ induced by 0.5% CSE at the indicated time points. Data was represented as mean ± SD. ^∗^*P* < 0.05, ^∗∗^*P* < 0.01, and ^∗∗∗^*P* < 0.001 compared to the control. CS: cigarette smoke; CSE: cigarette smoke extract.

To explore the role of the interaction between NOX4 and TGF-*β*_1_ in CS-induced COPD mice, the COPD mouse model exposed to CS with severe enlargement and destruction of alveoli in CS-exposed mice was firstly generated (Figure 2(a), *n* = 5 for both groups). Histological analysis showed that the mean linear intercept (MLI) in the lungs of the CS-exposed mice was significantly larger than that of the clean air-exposed mice (Figure 2(b), *n* = 5 for both groups), indicative of emphysematous lungs in the CS-exposed mice. The thickness of airway walls was quantified using the indicator of the small airway wall area/length (mm^2^/mm), which did not show statistical significance between CS-induced mice and control mice (*P* > 0.05) (Figure 2(e)). Immunoblotting assay results showed that compared with clean air-exposed mice, the expression of NOX4 and Smad3 phosphorylation was remarkably elevated in CS-exposed mice; the expression of TGF-*β*_1_ and *α*-SMA was increased but not statistically significant in CS-exposed mice (Figures 2(c) and 2(d), *n* = 6 for both groups). IHC showed that the immunoreactivity of NOX4, TGF-*β*, and *α*-SMA was increased in small airways of the CS-exposed mouse lungs as compared to that of the control group mice (Figure 2(e), *n* = 5 for both groups). In addition, the expression of *NOX4* and *TGF*-*β_1_* mRNAs was upregulated under stimulation with various concentrations of CSE (Figures 2(f) and 2(g)) for 24 h; and the increased levels of TGF-*β*_1_ protein induced by various concentrations of CSE for the indicated time in cell supernatants were assessed by ELISA (Figures 2(h) and 2(i)). These findings suggested that an increased level of NOX4 and TGF-*β*_1_ is found in the lungs of CS-induced emphysema mice and in HBSMC treatment with CSE.

### 3.4. NOX4 Is a Target of the Canonical TGF-*β* Pathway, along with *α*-SMA and Col I in HBSMCs

**Figure 3:**
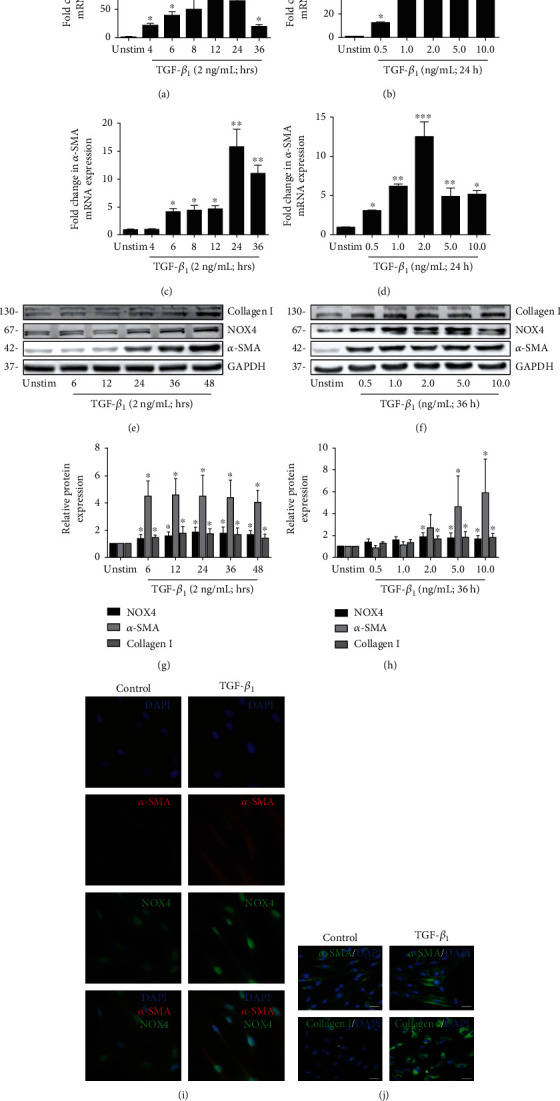
Effects of TGF-*β*_1_ on the expression of NOX4, *α*-SMA, and collagen type I in HBSMCs. (a, c, e) Cells were incubated with TGF-*β*_1_ (2 ng/mL) for the indicated time. (b, d, f) Cells were stimulated with various concentrations of TGF-*β*_1_ (0.5-10 ng/mL) for 24 h (mRNA levels) or 36 h (protein levels). (a, b) The mRNA levels of *NOX4* were determined by real-time Q-PCR. (c, d) The mRNA levels of *α*-*SMA* were determined by real-time Q-PCR. (e–h) The protein expression of NOX4, *α*-SMA, and collagen I was determined by Western blot. (i) Double immunofluorescence staining for colocalization of NOX4 (green) and *α*-SMA (red) in HBSMCs. DAPI (blue) labeled the nuclei. (j) Representative images of immunofluorescence staining of *α*-SMA (green) and collagen I (green) expression. Nuclei were labeled with DAPI (blue) in cells treated with/without 2 ng/mL TGF-*β*_1_ for 36 h. Data was represented as mean ± SD from three independent experiments and presented as mean ± SD. ^∗^*P* < 0.05, ^∗∗^*P* < 0.01, and ^∗∗∗^*P* < 0.001 compared to the unstimulated control. ^#^*P* < 0.05 compared to the unstimulated control. ^&^*P* < 0.05 compared to the unstimulated control.

Previously, we reported a correlation between increased expression of NOX4 and TGF-*β* and ASM remodeling of small airways in patients with COPD [11]. TGF-*β*_1_ contributes to airway remodeling by driving ECM production and deposition [31]. To further investigate the effect of TGF-*β*_1_ on the expression of NOX4, *α*-SMA, and Col I in HBSMCs, cells were treated with various concentrations of TGF-*β*_1_ for the indicated time points. The results showed that 2 ng/mL TGF-*β*_1_ significantly upregulated the levels of *NOX4 mRNA* in HBSMCs at 4 h, which peaked at 12 h and remained high at 36 h (Figure 3(a)), and the increased expression of *NOX4 mRNA* was induced by 0.5-10 ng/mL TGF-*β*_1_ for 24 h (Figure 3(b)). Simultaneously, the level of *α*-*SMA mRNA* was significantly increased after incubation with 2 ng/mL TGF-*β*_1_ at the indicated time (Figure 3(c)), and the upregulated expression of *α*-*SMA mRNA* was induced by 0.5-10 ng/mL TGF-*β*_1_ for 24 h in HBSMCs (Figure 3(d)). Furthermore, the abundance of NOX4, *α*-SMA, and Col I proteins was significantly increased after incubation with 2 ng/mL TGF-*β*_1_ for the indicated time (Figures 3(e) and 3(g)), and the increased expression of NOX4, *α*-SMA, and Col I proteins was induced by various concentrations TGF-*β*_1_ (Figures 3(f) and 3(h)). Double immunofluorescence staining demonstrated that the expression of NOX4 and *α*-SMA was markedly increased in TGF-*β*_1_ as compared with the control (Figure 3(i)), and the immunofluorescence result further demonstrated the increased expression of *α*-SMA and the excess production of Col I induced by TGF-*β*_1_ compared with the control (Figure 3(j)). Therefore, TGF-*β*_1_ was able to upregulate the expression of NOX4, along with the expression of *α*-SMA and the excess production of Col I.

### 3.5. NOX4-Derived ROS Was Required for TGF-*β*_1_-Induced *α*-SMA and Col I Expression in HBSMCs

**Figure 4:**
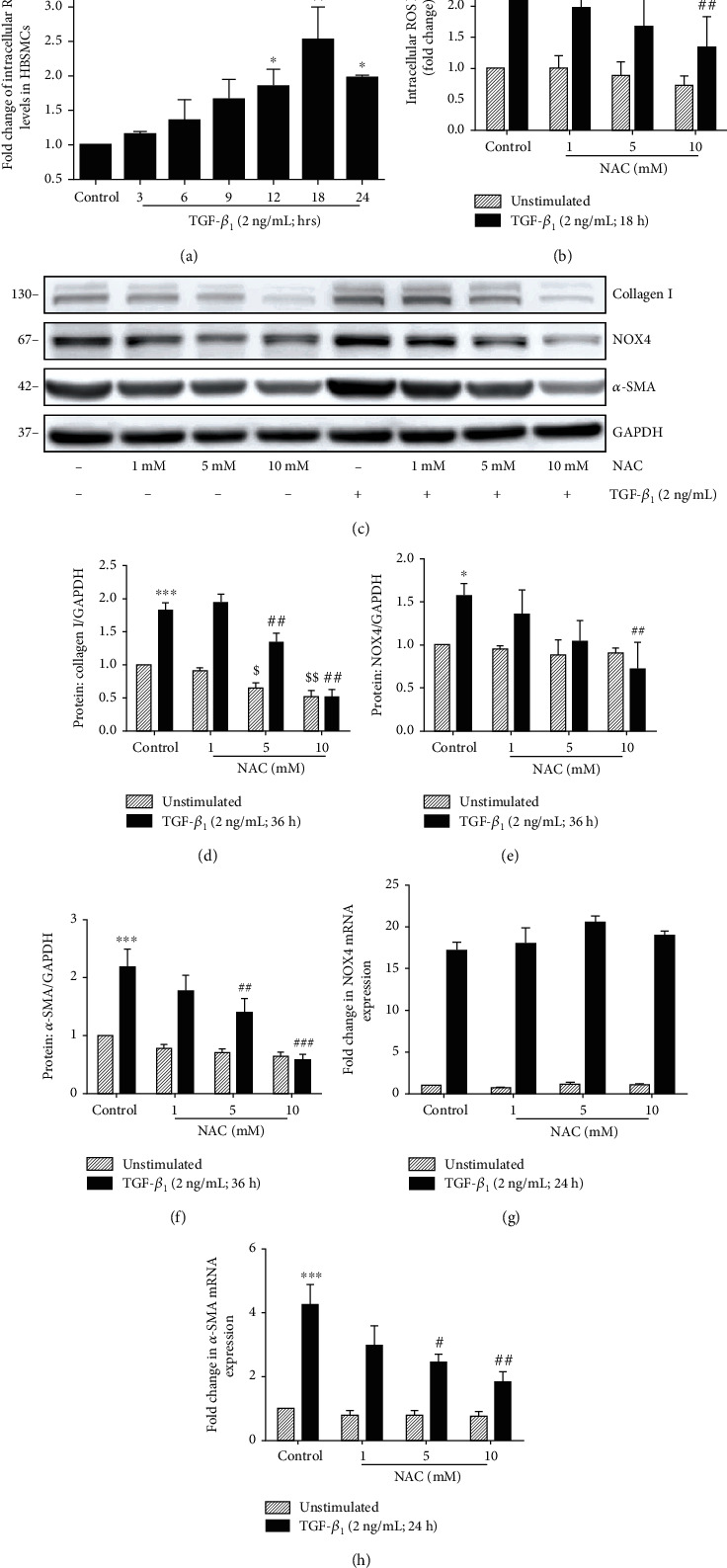
NAC scavenges ROS and attenuates TGF-*β*_1_-induced NOX4, *α*-SMA, and collagen type I expression in HBSMCs. (a, b) The intracellular ROS levels were determined by flow cytometry in HBSMCs. (a) Cells were incubated with 2 ng/mL TGF-*β*_1_ for the indicated time points. (b) Cells were pretreated with different concentrations of NAC (1-10 mM) for 1 h and incubated with 2 ng/mL TGF-*β*_1_ for 18 h. (c–h) The protein and mRNA expression of NOX4, *α*-SMA, and collagen I in HBMSCs. Cells were pretreated with NAC (1-10 mM) for 1 h, then incubated with 2 ng/mL TGF-*β*_1_ for 24 h (mRNA expression) or 36 h (protein expression); the protein expression of NOX4, *α*-SMA, and collagen I was determined by Western blot (c–f), and the mRNA expression of NOX4 and *α*-SMA was determined by real-time PCR (g, h). Data was represented as mean ± SD from three independent experiments and presented as mean ± SD. ^∗^*P* < 0.05, ^∗∗^*P* < 0.01, and ^∗∗∗^*P* < 0.001 compared to the unstimulated control. ^#^*P* < 0.05, ^##^*P* < 0.01, and ^###^*P* < 0.001 compared to the control with TGF-*β*_1_.

Oxidative stress is a critical amplifying mechanism in COPD, and the core factor is the increased ROS [5, 32]. Next, we accessed the intracellular ROS levels triggered by TGF-*β*_1_ by DCFH-DA staining in HBSMCs. As shown in Figure 4(a), TGF-*β*_1_ increases the intracellular ROS to a maximal level of 2.5-fold at 18 h. To determine the effect of ROS on HBSMCs, we pretreated the cells with NAC, a scavenger of ROS (1–10 mM), for 1 h before the TGF-*β*_1_ treatment, and then detected that the levels of intracellular ROS and the protein and mRNA levels of NOX4, *α*-SMA, and Col I in HBSMCs. The results showed that NAC (5 and 10 mM) significantly reduced the intracellular ROS (Figure 4(b)), downregulated the expression of NOX4, *α*-SMA, and Col I proteins (Figures 4(c)–4(f)) and the mRNA level of *α*-SMA (Figure 4(h)), but did not alter the expression of NOX4 mRNA (Figure 4(g)). Consequently, these results indicated that ROS mediated the TGF-*β*_1_-induced expression of NOX4, *α*-SMA, and Col I in HBSMCs.

**Figure 5:**
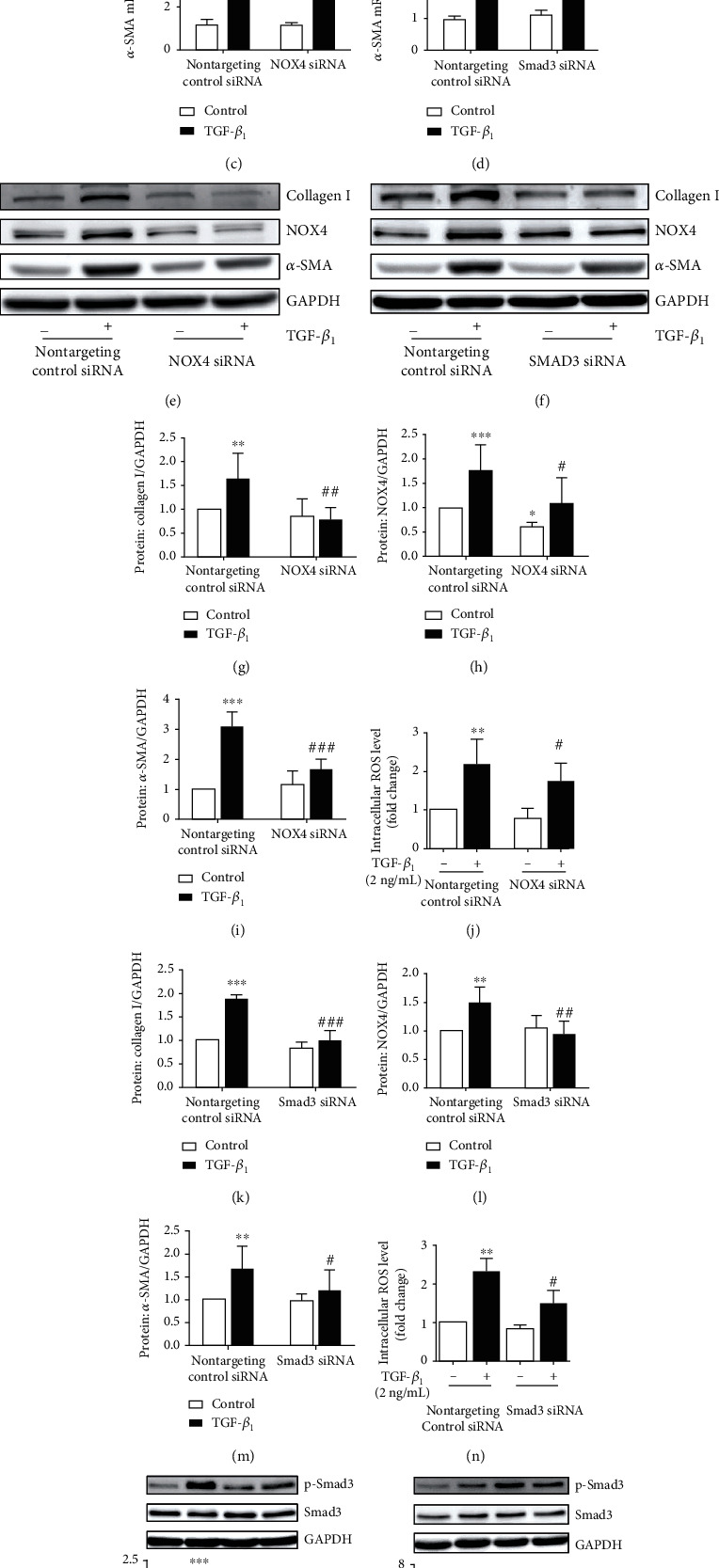
The effect of NOX4 siRNA and Smad3 siRNA on TGF-*β*_1_-induced expression of NOX4, *α*-SMA, and collagen I and the ROS production in HBSMCs. Cells were transfected with either nontargeting (control) siRNA or *NOX4* siRNA and Smad3 siRNA for 24 h and then incubated with/without TGF-*β*_1_ (2 ng/mL) for 18 h (ROS levels), 24 h (mRNA levels), or 36 h (protein levels). (a–d) The mRNA level of NOX4 (a, b) and *α*-SMA (c, d) was analyzed by real-time PCR. (e, f) The protein expression of NOX4, *α*-SMA, and collagen I was analyzed by Western blot. (g–n) The immunoreactivity of NOX4 (h, l), *α*-SMA (i, m), and collagen I (g, k) was measured by ImageJ software. (j, n) Intracellular ROS levels were detected by flow cytometry measuring DCF mean fluorescence intensity and fold change of intracellular ROS levels. (o, p) Cells were transfected with siRNA for 24 h or pretreated with NAC (10 mM) for 1 h. Treatment with TGF-*β*_1_ for 0.5 h. The effect of Smad3 siRNA (j) and NAC (k) on the TGF-*β*_1_-induced level of basal and phosphorylation of Smad3 protein. Data was represented as mean ± SD from three independent experiments and presented as mean ± SD. ^∗∗^*P* < 0.01, ^∗∗∗^*P* < 0.001, ^#^*P* < 0.05, ^##^*P* < 0.01, and ^##^*P* < 0.001 compared to the control siRNA with/without TGF-*β*_1_.

Next, we explored the correlation between NOX4 and intracellular ROS as well as the effect of oxidative stress on ECM deposition and HBSMC proliferation. First, the efficiency of the knockdown of four duplexes of human NOX4 siRNA sequences was evaluated by real-time PCR and Western blot in HBSMCs (Suppl. Fig. S1A-B) and NOX4-4 siRNA was selected as the target sequence of the knockdown of the *NOX4* gene (Figures 5(a), 5(e), and 5(h)). Next, we transfected NOX4 siRNA to the knockdown of the *NOX4* gene, followed by treatment with or without TGF-*β*_1_ (2 ng/mL) for 24 or 36 h. We found that the knockdown of the NOX4 gene significantly suppressed the TGF-*β*_1_-induced protein and mRNA expression of *α*-SMA (Figures 5(b), 5(e), and 5(i)) and inhibited the protein expression of Col I (Figures 5(e) and 5(g)). Furthermore, the flow cytometry confirmed that TGF-*β*_1_-induced production of intracellular ROS was inhibited by *NOX4* siRNA transfection (Figure 5(j)). These results suggested that NOX4 mediated TGF-*β*_1_-induced *α*-SMA and Col I expression and the production of ROS in HBSMCs.

Smad is activated by TGF-*β*_1_ enrolled in various signal pathways, and Smad3 is the key downstream transcript factor of the TGF-*β*_1_ signaling cassette [25, 30]. In this study, we observed that TGF-*β*_1_ activated Smad3 phosphorylation at 30 min, which decreased gradually over a 24 h period (Suppl. Fig. S2A). To confirm the involvement of Smad3 in TGF-*β*_1_-induced *NOX4*, *α*-*SMA*, and *Col I* gene expression and the production of ROS in HBSMCs, the Smad3 expression was knocked down by transfection of Smad3 siRNA in HBSMCs. The result of Western blot showed that the abundance of Smad3 protein was reduced by 84.53 ± 1.67% after the transfection of Smad3 siRNA as compared with cells transfected with the control siRNA, and the knockdown blocked the TGF-*β*_1_-induced phosphorylation of Smad3 (Suppl. Fig. S2B). The specific knockdown of Smad3 significantly decreased the TGF-*β*_1_-induced mRNA expression of *NOX4* (Figure 5(c)) and *α*-*SMA* (Figure 5(d)), suppressed the expression of NOX4, *α*-SMA, and Col I proteins (Figures 5(f) and 5(k)–5(m)), and inhibited TGF-*β*_1_-induced intracellular ROS production in HBSMCs (Figure 5(n)). As to the previous report, the phosphorylation of Smad3 was mediated by ROS, but the effect of NOX4 on the phosphorylation of Smad3 was unclear. Our study showed that NOX4 siRNA (Figure 5(o)) and NAC treatment (Figure 5(p)) significantly inhibited the TGF-*β*_1_-mediated phosphorylation of Smad3. Consequently, these results suggested that TGF-*β*_1_-induced NOX4 and *α*-SMA expression and Col I synthesis of HBSMCs were dependent on Smad3, and the production of ROS had a positive feedback effect on the phosphorylation of Smad3.

### 3.6. ROS-Mediated Activation of p38MAPK/Akt Signaling Was Responsible for TGF-*β*_1_-Induced *α*-SMA and Col I Expression

**Figure 6:**
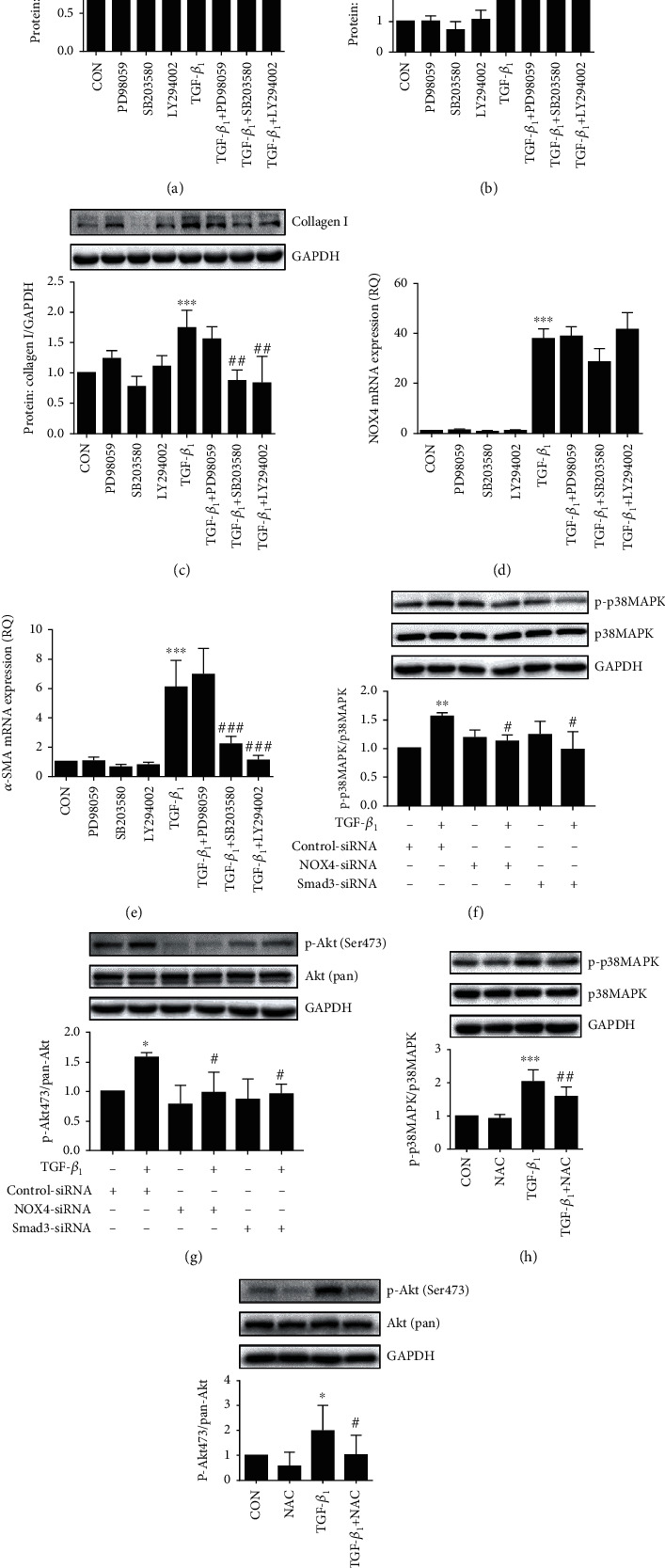
ROS-mediated activation of p38MAPK/Akt signaling was responsible for TGF-*β*_1_-induced *α*-SMA and Col I expression. (a–e) Cells were pretreated with different signaling pathway inhibitors PD98059 (10 mM), SB203580 (10 mM), and LY294002 (10 mM) before exposure to TGF-*β*_1_ (2 ng/mL). (a–c) The impact of inhibitors on the protein expression of NOX4 (a), *α*-SMA (b), and collagen I (c) after treatment with 2 ng/mL TGF-*β*_1_ for 36 h, as determined by Western blot. (d, e) The impact of inhibitors on the mRNA levels of NOX4 (d) and *α*-SMA (e) after incubation with 2 ng/mL TGF-*β*_1_ for 24 h; the mRNA levels were determined by real-time PCR. (f, g) Cells were transfected with scramble control, NOX4, and Smad3 siRNA for 24 h and treated with 2 ng/mL TGF-*β*_1_ for 12 h; the protein levels of p-p38MAPK (f) and p-Akt473 (g) were determined by Western blot. (h, i) Cells were pretreated with NAC (10 mM) before exposure to TGF-*β*_1_ (2 ng/mL) for 12 h; the protein levels of p-p38MAPK (h) and p-Akt473 (i) were detected by Western blot. Data was represented as mean ± SD from three independent experiments and presented as mean ± SD. CON: control. For (a–e) and (h, i): ^∗^*P* < 0.05, ^∗∗^*P* < 0.01, and ^∗∗∗^*P* < 0.001 compared to the control; ^#^*P* < 0.05 and ^##^*P* < 0.01 compared to TGF-*β*_1_. For (f, g): ^∗^*P* < 0.05, ^∗∗^*P* < 0.01, and ^#^*P* < 0.05 compared to the control siRNA with/without TGF-*β*_1_.

As a Smad3-independent pathway, the activation of the MAPK or Akt pathway is known to participate in the effect of TGF-*β*_1_-induced cell differentiation and ECM synthesis in airway structure cells. We further detected the activation of MAPK and Akt pathways in response to TGF-*β*_1_. We found that TGF-*β*_1_ increased phosphorylation of ERK, p38MAPK, and p-Akt473 in a time-dependent manner but has no effect on p-Akt308 in HBSMCs (Suppl. Fig. S3A-D). The pretreatment with various inhibitors PD98059 (10 *μ*M), SB203580 (10 *μ*M), and LY294002 (10 *μ*M) for 1 h before TGF-*β*_1_ treatment has no effect on the expression of NOX4 in protein (Figure 6(a)) and mRNA levels (Figure 6(d)). PD98059, an ERK1/2 MAPK inhibitor, had no effect on the expression of *α*-SMA and collagen I in mRNA and protein levels induced by TGF-*β*_1_ in HBSMCs (Figures 6(b), 6(c), and 6(e)). However, SB203580 (p38MAPK inhibitor) and LY294002 (Akt inhibitor) notably suppressed the TGF-*β*_1_-increased expression of *α*-SMA and collagen I in HBSMCs (Figures 6(b), 6(c), and 6(e)). To clarify the detailed correlation between the activated signal molecules (p38MAPK and p-Akt473) and NOX4 or Smad3, we further transfected specific *NOX4* or *Smad3* siRNA before TGF-*β*_1_ treatment. The results showed that *NOX4* or *Smad3* siRNA could block the phosphorylation of p38MAPK and Akt (Figures 6(f) and 6(g)). The addition of NAC (10 mM) also inhibited the phosphorylation of p38MAPK and Akt (Figures 6(h) and 6(i)). Taken together, these data suggested that TGF-*β*_1_-induced *α*-SMA and *α*-collagen I production in HBSMCs was mediated by the Smad3-dependent NOX4-activated p38MAPK or Akt signaling pathway.

## 4. Discussion

Our previous works revealed an increased expression of NOX4 and TGF-*β* in the ASM of the small airway of the human COPD lung, which was positively correlated with the severity of airflow limitation [11]. In the present study, we found that the expression of NOX4 and *α*-SMA was markedly elevated in the small airway of COPD patients and CS-induced emphysema mice. These data imply that NOX4 may be a key player in the ASM remodeling during the course and pathogenesis of COPD. In addition, we further revealed that the feedback loop of NOX4-derived ROS/Smad was involved in TGF-*β*_1_-mediated HBSMC differentiation and collagen I production. Mechanistically, the NOX4-derived ROS activated p38MAPK and Akt signaling and substantially mediated TGF-*β*_1_-induced HBSMC differentiation and collagen I production.

Interestingly, in this study, we observed the small airway wall thickness in COPD patients compared with the control group, but we failed to observe the airway wall thickness in CS-induced COPD mice. In addition, we observed the elevated expression of *α*-SMA and TGF-*β*_1_ in small airway walls in COPD patients but not significantly in CS-induced mice. However, we observed the remarkably elevated expression of the phosphorylated level of Smad3 in CS-induced mice. According to Tam et al.'s report [33], they focus on the impact of female sex hormones on chronic cigarette smoke-induced airway remodeling and emphysema in a mouse model of COPD; their results showed that small airway wall remodeling was increased in females but not males and was associated with increased distal airway resistance and activation of TGF-*β*_1_. In our study, the C57BL/6 mice were selected as CS-induced COPD mice and control mice were all male mice; this may be the reason why we did not observe the airway remodeling in the CS-induced mouse model. Based on the above conflicting data, we reasoned that the discrepancy of the expression of TGF-*β* reported in different studies might be caused by different sample collections and the proportion of male and female subjects and the exposure factor or duration. These results and our findings merit further investigation into the roles of TGF-*β* activities in the pathogenesis of COPD. In addition, the cellular location of NOX4 was different in the human lung biopsy determined by IHC assay and the primary cells accessed by IF assay. We reasoned that the discrepancy might be caused by means of staining and/or the tissues used for this particular antibody.

TGF-*β*_1_ is a growth factor and cytokine involved in the pathogenesis of asthma and other airway diseases [30]. It is elevated in the lungs of COPD patients [29] and plays a role in airway remodeling by enhancing ASM proliferation and ECM deposition [34, 35]. In COPD patients, there was an elevated expression of NOX4 and TGF-*β*_1_ in the small airways accompanied with small airway remodeling, which was consistent with an elevated expression of NOX4 and phosphorylation of Smad3 in the lungs of CS-induced mice. This result was divergent from the finding reported in a previous study by Hoang et al. [36], whose data showed that CS reduced the level of TGF-*β*_1_ and increased that of TGF-*β*_2_ after 12 weeks of smoke exposure in rats. In a clinical study, Llinàs et al. [37] observed a decreased *TGF*-*β_1_ mRNA* expression in the peripheral lungs of patients with severe stable COPD as compared to the control nonsmoking subjects. In contrast, Vignola et al. [38] demonstrated increased TGF-*β*_1_ immunostaining in the bronchial biopsy samples of patients with chronic bronchitis as compared to the control young nonsmoking subjects.

A previous cross-sectional study demonstrated that the progression of COPD is associated with increased inflammation and an abnormal tissue repair process [4]. In COPD, it is believed that changes in major lung ECM components are involved in the loss of elasticity during emphysema progression [5, 6]. Notably, there is no consensus on the number of ECM components in the airways and parenchyma in COPD patients [39]. For example, Annoni et al. observed a decreased abundance of elastic fibers, collagen subtype I, and versican in small and large airways of moderate COPD patients [40]. However, no significant difference in the collagen biomarker levels was determined in lungs between healthy smokers, healthy nonsmokers, and COPD patients in another study by Bihlet et al. [39]. Several lines of studies demonstrated in vitro that ASM cells from COPD patients stimulated with cigarette smoke (CS) extract have higher deposition of collagen type VIII alpha I, but there were no differences in the deposition of collagen V and fibronectin [41, 42]; ASM is the main effector in ARM, which secretes ECM components and, in turn, is functionally responsive to the ECM composition [40]. In the present study, we found that the Col I synthesis in ASM is markedly elevated by TGF-*β* stimulation in a time- and concentration-dependent manner. These data imply that ASM plays a major role in ECM and remodeling via Col I degradation and formation. A previous study showed that there are differences in the ECM fiber remodeling patterns between experimental models of emphysema compared to different experimental models of emphysema. Lopes et al. showed that there was an increase in collagen III but not in collage I in both the CS model and the porcine pancreatic elastase model of emphysema [43]. In COPD patients, Harju et al. showed that the amounts of precursors of collagen I and III were increased in smokers and stage I-II COPD but tended to decline in either large or small airways in stage IV COPD [42]. Annoni et al. showed a decrease in elastic fibers, collagen subtype I, and versican, in small and large airways, associated with a higher fibronectin fractional area; the divergence of results may be attributed to the different ECM components and limitations of the lung sample obtained from biopsy or resection [40].

The imbalance of oxidative stress responses in ASM has been shown in patients with COPD and chronic airway inflammation, where it affects the function of ASM [10]. Notably, the subcellular localization and distribution of NADPH oxidases are cell type-dependent [44, 45]. In ASM cells, NOX4 is the main NOX isoform and plays a critical role in ASM remodeling. NOX4 was elevated in the ASM layer of small airways in both COPD patients [11] and CS-exposed mice. In addition, correlation analysis demonstrated that the level of NOX4 in ASM was inversely associated with the pulmonary function but positively correlated with the abundance of ECM markers and TGF-*β* in the small airways of COPD lungs [11]. In this context, NOX4 might be the main regulator in ARM where it was regulated by TGF-*β* signaling [11]. Indeed, TGF-*β*_1_ was able to induce *α*-SMA expression and ECM protein production in ASM cells. This effect could be reversed by ROS scavenger NAC and NOX4 silencing. These data imply that oxidative stress was elevated in ASM in the pathophysiological process of COPD, which is tightly correlated with the prooxidant effect of TGF-*β*.

Emerging evidence indicated that NADPH oxidases as downstream mediators in TGF-*β* induced profibrotic effects [43]; there is also evidence suggesting that redox pathways may regulate TGF-*β*/Smad signaling in a feed-forward manner [43]. ROS molecules may have critical roles in facilitating TGF-*β*-induced Smad2/3 activation [43]. The mechanism of the enhancing effect of ROS on TGF-*β*-induced Smad phosphorylation is currently unclear. In this study, we showed that the overproduction of NOX4-derived ROS could be abolished by siRNA to *Smad3*. In addition, the TGF-*β*-induced Smad2/3 phosphorylation could be blocked by NAC. These data imply that redox pathways may regulate TGF-*β*/Smad signaling in a feed-forward manner. Moreover, the activation of MAPK is identified as a critical molecular mechanism for ARM in the small airway [46].

Phosphorylation of Smad3 by TGF is important for the induction of NOX4. Martin-Garrido et al. reported that NOX4-mediated activation of p38MAPK leads to the phosphorylation and activation of serum response factor (SRF), increases SRF-dependent transcriptional activity, and increases smooth muscle *α*-actin (SMA) expression [47]. It was previously reported that TGF-*β* may activate other signaling pathways including the mitogen-activated protein kinase (MAPK) members, such as c-Jun N-terminal kinase (JNK) and p38. Additionally, JNK and p38 may in turn enhance the transcriptional activities of Smad proteins by direct phosphorylation of Smad3 or indirectly promoting Smad3 association with the transcriptional coactivator p300 [48]. Jiang et al. found that both JNK and p38 can be activated by ROS in the cytoplasm [49]. Although our data showed that TGF-*β*_1_ significantly upregulated the levels of NOX4 mRNA and NOX4 protein in HBSMCs at 4 h and at 24 h, respectively, the increase of NOX4 protein was lagged to the activation of Smad3, p38MAPK, and Akt. We speculated there was a crosslink between the Smad3-dependent pathway and the p38MAPK or Akt signaling pathway and this crosslink might relate to ROS. The divergence of results may be attributed to the time point we selected and may need further investigation.

**Figure 7:**
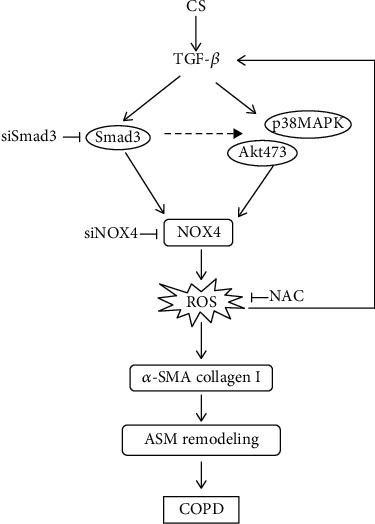
Schematic representation of Smad3-NOX4-derived ROS-mediated p38MAPK/Akt signaling in TGF-*β*_1_-induced ECM production of HBSMCs. We previously demonstrated an increased expression of NOX4 and TGF-*β*_1_ with the severity of remodeling in ASM of the small airway of the COPD lung, which was inversely associated with the pulmonary function. The findings in the present study demonstrated that NOX4 mediated TGF-*β*_1_-induced ECM protein synthesis and differentiation in HBSMCs; NOX4-derived ROS in response to TGF-*β*_1_ may activate the p38MAPK or Akt pathway, resulting in the differentiation and ECM protein synthesis based on the Smad3 canonical pathway. And NAC, a scavenger of ROS, attenuates HBSMC remodeling by scavenging intracellular ROS to inhibit NOX4 expression and block the Smad3 and MAPK pathways. Current findings indicated that NOX4 is critical for modulation in TGF-*β*_1_-induced ECM synthesis in HBSMCs, which contributes to airway remodeling in the pathogenesis of COPD, and the antioxidant (NAC) might serve as a valuable therapeutic strategy.

Taken together, the present study suggested that TGF-*β*_1_ increased the oxidative stress by regulating NOX4-derived generation of ROS via phosphorylation of Smad3 and subsequently activating MAPK/Akt transduction (Figure 7). This overwhelming NOX4 further promoted the differentiation and ECM production in HBSMCs and effectuated the ARM in COPD lungs. These data thus implied that NOX4 signaling might be a promising target for developing strategies and agents for COPD prevention and treatment, which also provide an insight into the underlying mechanism of NOX4 in airway remodeling.

## Figures and Tables

**Table 1:** Demographics of COPD patients and healthy cohorts.

Demographics	Control (*n* = 21)	COPD (*n* = 28)	*P* values
Age	55.43 ± 7.65	59.68 ± 9.43	0.10
Gender (male/female)	9/12	20/8	
WBC (×10^9^/L)	5.34 ± 1.08	6.72 ± 2.28	0.01
Neutrophils (×10^9^/L)	2.87 ± 0.71	4.20 ± 1.84	≤0.01
Lymphocytes (×10^9^/L)	1.87 ± 0.58	1.76 ± 0.52	0.48
NLR	1.72 ± 0.94	2.56 ± 1.22	0.01
FEV1	2.70 ± 0.68	2.18 ± 0.72	0.02
FVC	3.40 ± 0.86	3.55 ± 0.88	0.55
FEV1/FVC%	79.74 ± 3.31	60.95 ± 10.57	≤0.01
FEV1%pred	101.95 ± 19.60	82.09 ± 26.53	0.04

FEV1: forced expiratory volume in one second; FVC: forced vital capacity; NLR: neutrophil-lymphocyte ratio; WBC: white blood cell.

## Data Availability

The datasets used and/or analyzed during the current study are available from the corresponding authors on reasonable request.

## Abbreviations

ASM: Airway smooth muscle
AEC: Airway epithelial cell
ARM: Airway remodeling
COPD: Chronic obstructive pulmonary disease
Col I: Collagen I
CSE: Cigarette smoke extract
ECM: Extracellular matrix
FEV1: Forced expiratory volume in one second
FN: Fibronectin
LAP: Latency-associated peptide
FVC: Forced vital capacity
NAC: N-Acetylcysteine
NADPH: Nicotinamide adenine dinucleotide phosphate
NLR: Neutrophil-lymphocyte ratio
p38MAPK: p38 mitogen-activated protein kinase
ROS: Reactive oxygen species
TGF-*β*_1_: Transforming growth factor *β*_1_
WBC: White blood cell.

## Supplementary Materials

Supplementary Materials S1: supplemental figures. S1.1. Suppl. Fig. S1: efficiency of NOX4 silencing induced by TGF-*β*_1_ in HBSMCs. (A) HBSMCs were transfected with individual NOX4 siRNA for 48 h; the mRNA expression of *NOX4* was determined by Q-PCR. (B) HBSMCs were transfected with individual NOX4 siRNA for 24 h, then treated with 2 ng/mL TGF-*β*_1_ for 48 h; the expression of NOX4 protein was determined by Western blot. Data was presented as mean ± SD. ^∗∗∗^*P* < 0.001 compared to the control. S1.2. Suppl. Fig. S2: the expression of the phosphorylation of Smad3 induced by TGF-*β*_1_ in HBSMCs. (A) The phosphorylated levels of Smad3 induced by 2 ng/mL TGF-*β*_1_ at the indicated time. (B) The total and phosphorylated levels of Smad3 induced by TGF-*β*_1_ after transfected with Smad3 siRNA. Data was presented as mean ± SD. ^∗∗∗^*P* < 0.001 compared to NC without TGF-*β*_1_ treatment. NC: nontargeting control siRNA. S1.3. Suppl. Fig. S3: the phosphorylated levels of ERK1/2, p38MAPK, and Akt473 induced by TGF-*β*_1_ in HBSMCs. HBSMCs were stimulated with 2 ng/mL TGF-*β*_1_ at the indicated time; the expression of total and phosphorylated levels of ERK1/2, p38MAPK, and Akt was determined by Western blot. (A) The expression of ERK1/2 and p-ERK1/2. (B) The expression of Akt and p-Akt473. (C) The expression of p38MAPK and p-p38MAPK. (D) The fold change of phosphor-protein compared to the total protein. Data was presented as mean ± SD. ^∗^*P* < 0.05, ^#^*P* < 0.05, ^&^*P* < 0.05, ^∗∗^*P* < 0.01, and ^##^*P* < 0.05 compared to the control. S2: detailed methods. Described the detailed information on the reagents and methods employed in this study.
