# Supplementary material for: NOX4-Derived ROS Promotes Collagen I Deposition in Bronchial Smooth Muscle Cells by Activating Noncanonical p38MAPK/Akt-Mediated TGF-β Signaling
Source: Oxid Med Cell Longev. 2021 Mar 19;2021:6668971. doi: 10.1155/2021/6668971 (PMC8007363; doi:10.1155/2021/6668971)
Supplement: Supplementary Materials — S1: supplemental figures. S1.1. Suppl. Fig. S1: efficiency of NOX4 silencing induced by TGF-β1 in HBSMCs. (A) HBSMCs were transfected with individual NOX4 siRNA for 48 h; the mRNA expression of NOX4 was determined by Q-PCR. (B) HBSMCs were transfected with individual NOX4 siRNA for 24 h, then treated with 2 ng/mL TGF-β1 for 48 h; the expression of NOX4 protein was determined by Western blot. Data was presented as mean ± SD. ∗∗∗P < 0.001 compared to the control. S1.2. Suppl. Fig. S2: the expression of the phosphorylation of Smad3 induced by TGF-β1 in HBSMCs. (A) The phosphorylated levels of Smad3 induced by 2 ng/mL TGF-β1 at the indicated time. (B) The total and phosphorylated levels of Smad3 induced by TGF-β1 after transfected with Smad3 siRNA. Data was presented as mean ± SD. ∗∗∗P < 0.001 compared to NC without TGF-β1 treatment. NC: nontargeting control siRNA. S1.3. Suppl. Fig. S3: the phosphorylated levels of ERK1/2, p38MAPK, and Akt473 induced by TGF-β1 in HBSMCs. HBSMCs were stimulated with 2 ng/mL TGF-β1 at the indicated time; the expression of total and phosphorylated levels of ERK1/2, p38MAPK, and Akt was determined by Western blot. (A) The expression of ERK1/2 and p-ERK1/2. (B) The expression of Akt and p-Akt473. (C) The expression of p38MAPK and p-p38MAPK. (D) The fold change of phosphor-protein compared to the total protein. Data was presented as mean ± SD. ∗P < 0.05, #P < 0.05, &P < 0.05, ∗∗P < 0.01, and ##P < 0.05 compared to the control. S2: detailed methods. Described the detailed information on the reagents and methods employed in this study. [file 6668971.f1.docx]

**Supplemental materials**

**S1. Supplemental Figures**

**
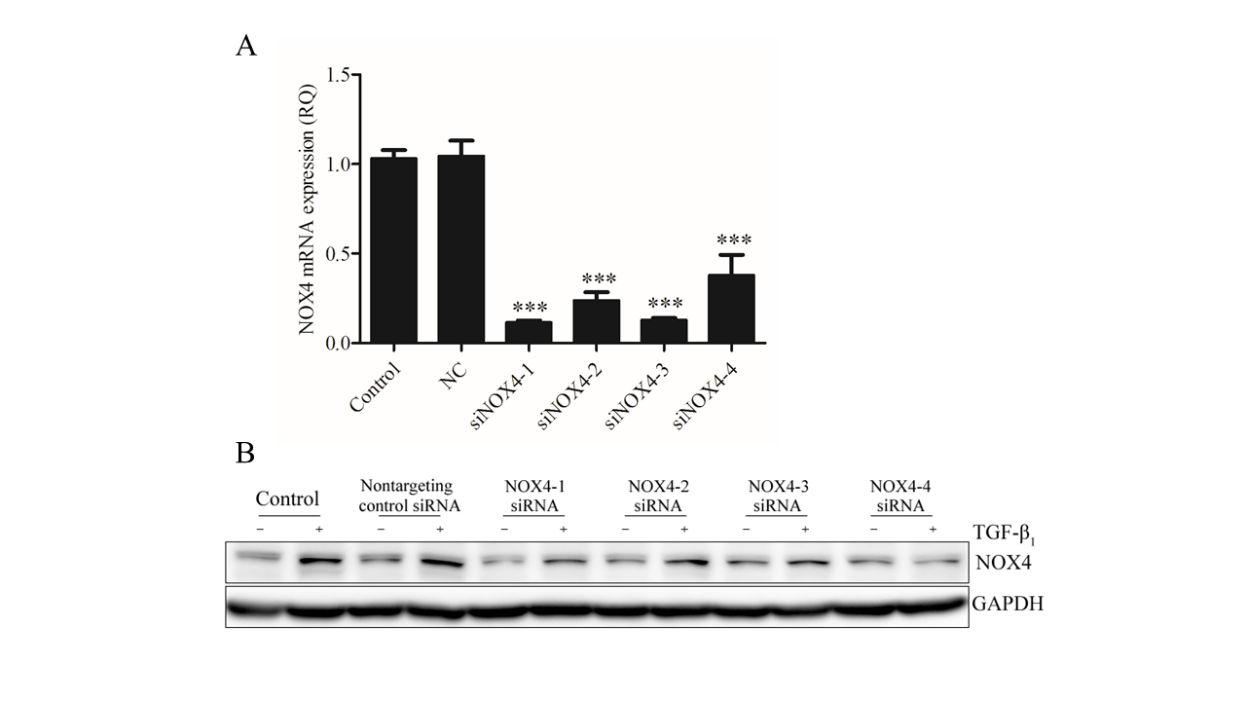
**

**Suppl. Fig. S1: Efficiency of NOX4 silence induced by TGF-β_1_ in HBSMC.** A: HBSMCs were transfected with individual Nox4-siRNA for 48h, the mRNA expression of *NOX4* was determined by Q-PCR; B. HBSMCs were transfected with individual NOX4 siRNA for 24h, then treated with 2ng/mL TGF-β_1_ for 48h, the expression of NOX4 protein was determined by western blot. Data represent Mean±SD from three independent experiments and presented as Mean±SD, ***P<0.001 compared to control.

**
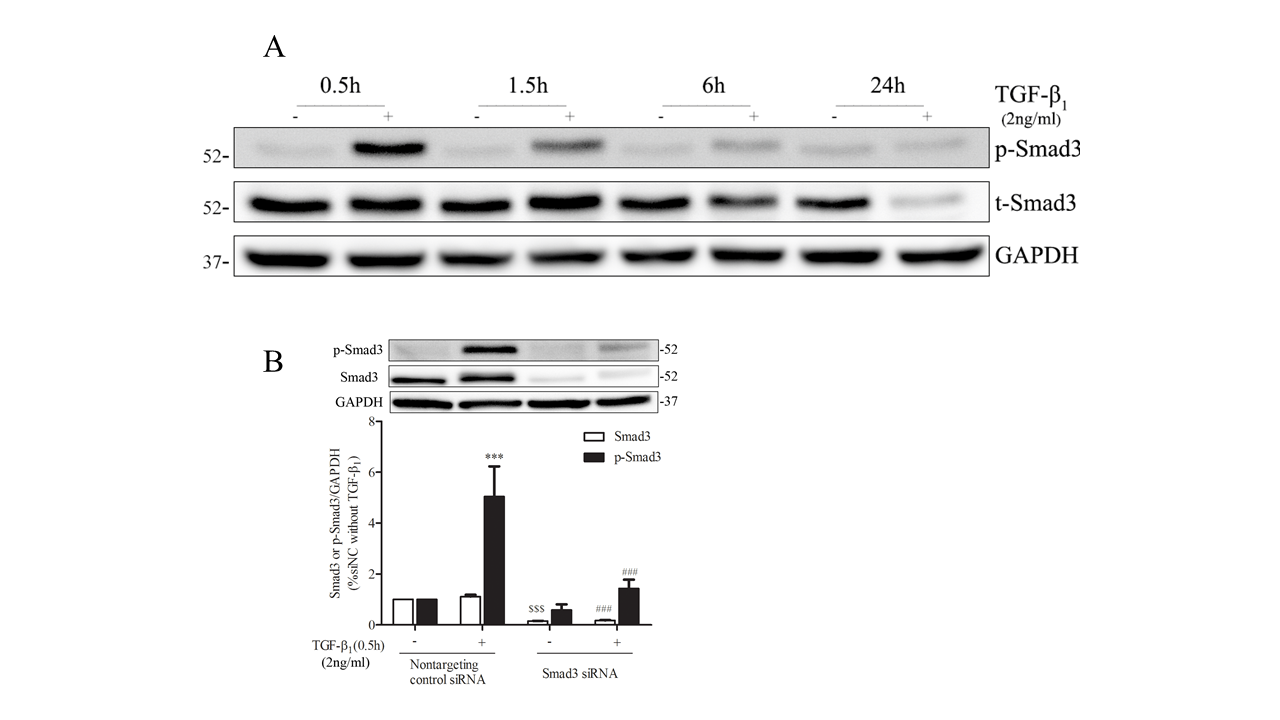
**

**Suppl. Fig. S2: The expression of the phosphorylation of SMAD3 induced by TGF-β_1_ in HBSMC.** A. The phosphorylated levels of smad3 induced by 2ng/mL TGF-β_1_ at indicated time. B The total and phosphorylated levels of smad3 induced by TGF-β_1_ after transfected with SMAD3-siRNA. Data are representative of three independent experiments and presented as Mean±SD, ***P<0.001 compared to NC without TGF-β_1_ treatment. NC: scramble control siRNA.

**
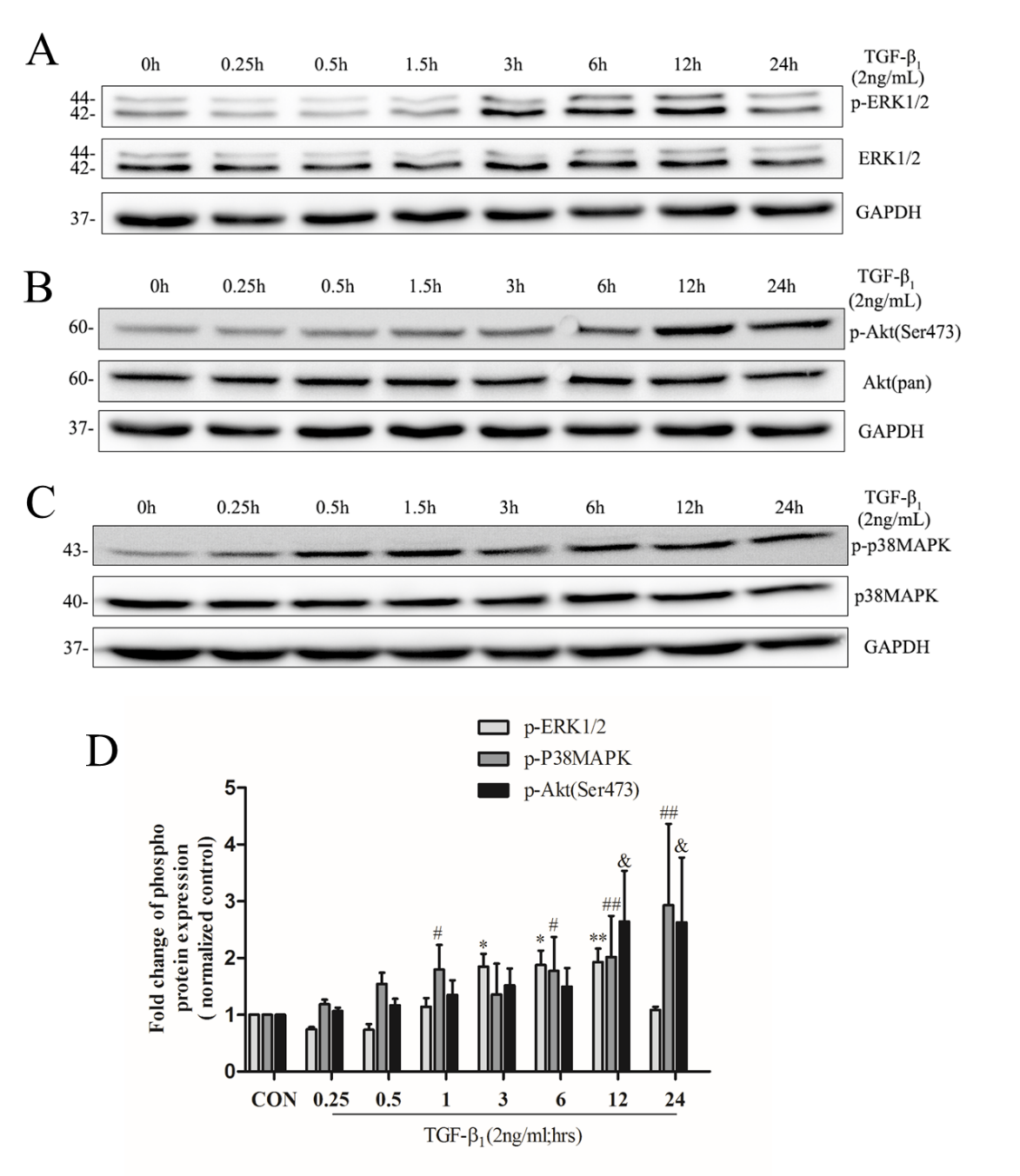
**

**Suppl. Fig. S3: The phosphorylation levels of ERK1/2, p38MAPK and Akt473 induced by TGF-β_1_ in HBSMCs.** HBSMCs were stimulated with 2ng/mL TGF-β_1_ at indicated time, the expression of total and phosphorylated levels of ERK1/2, p38MAPK and Akt was determined by western blot. A: The expression of ERK1/2 and p-ERK1/2; B: The expression of Akt and p-Akt473; C: The expression of p38MAPK and p-p38MAPK; D The fold-change of phosphor-protein compared to the total protein.Data are representative of three independent experiments and presented as Means±SD, *P<0.05 ^#^P<0.05 &P<0.05; **P<0.01 ^##^P<0.05 compared to Control.

**S2. Supplemental Materials and Methods**

**Reagents**

Human recombinant TGF-β_1_ was obtained from R&D Systems Europe (Abingdon, UK). N-acetyl-L-cysteine (NAC), the PI3K inhibitor LY294002 was purchased from Sigma-Aldrich (St. Louis, MO). The rabbit polyclonal, mouse monoclonal anti-α-SMA, and rabbit polyclonal anti-Collagen I was purchased from Abcam (Cambridge, UK). Rabbit anti-NADPH oxidase 4 (NOX4) antibody was obtained from Novus Biologicals (Littleton, CO, USA). The p38MAPK inhibitor SB203580, ERK1/2 inhibitor PD98059, and antibodies against phospho-p42/p44MAPK, p42/44MAPK, phospho-p38MAPK, p38MAPK, phospho-Smad3, Smad3, phospho-Akt(Ser473 and Thr308) and pan-Akt were purchased from Cell Signaling Technology (Danvers, MA). H_2_DCFH-DA was obtained from Molecular Probes (Eugene, OR). Smooth Muscle Cell Medium (SMCM, Cat.No.1101), fetal bovine serum (FBS Cat.No.0010), smooth muscle cell growth supplement (SMCGS Cat.No.1152) and penicillin/streptomycin solution (P/S Cat.No.0503) were purchased from ScienCell^TM^. The detail information of antibodies were shown in Suppl. Table S1.

**Suppl. Table S1. the detail information of antibodies**

| Antibody | catalogue numbers | Company |  |
| --- | --- | --- | --- |
| NOX4 | NB110-58849 | NOVUS | WB (1:500) IHC/IF（1:100） |
| α-SMA | ab5694 | Abcam | WB (1:1000) IHC (1:500) IF (1:200) |
| TGF-β1 | ab27969 | Abcam | WB (1:1000) |
| Collagen I | ab34710 | Abcam | WB (1:2000) IF (1:400) |
| SMAD3 | #9523 | Cell Signaling Technology | WB (1:1000) |
| P-SMAD3 | #9520 | Cell Signaling Technology | WB (1:1000) |
| ERK1/2 | #9170 | Cell Signaling Technology | WB (1:2000) |
| P-ERK1/2 | #4377 | Cell Signaling Technology | WB (1:1000) |
| p38MAPK | #8690 | Cell Signaling Technology | WB (1:1000) |
| p-p38MAPK | #9152 | Cell Signaling Technology | WB (1:1000) |
| pan-Akt | #4685 | Cell Signaling Technology | WB (1:1000) |
| Akt473 | #4060 | Cell Signaling Technology | WB (1:1000) |

**Animal experimental design**

Mice were exposed to cigarette smoke as described before[47]. Briefly, 12 mice were divided into control (clean air exposure) and cigarette smoke (CS) exposure groups. The mice in the CS group were exposed whole body to cigarettes (Red Rose: 13mg of tar/pack and 10mg of nicotine/pack), four 1-h period/day with a 20min smoke-free interval every 1-h period in a closed fume box for 6 days/week for 6 months. The control mice were exposed to clean air.

**Histology**

**Animal model:** After 6 months exposure, while the mice under anesthesia, the left lung was perfused with 4% formaldehyde at a constant pressure of 25cm H_2_O, and immersed in 4% formaldehyde for fixation at 4 ℃ for 24 h before paraffin embedding. Then the lung tissues sliced into 4.0-μm-thick sections for hematoxylin and eosin staining (HE-staining) or immunohistochemistry (IHC) analysis, as described previously[46, 48]. All tissue sections were deparaffinized and rehydrated through graded alcohol solution. **HE-staining**: ten fields were randomly captured from the staining sections that scanned at 100x magnification with Aperio-CS2 image capture device (Leica Biosystems, Germany). The enlargement of alveolar spaces was quantified by the measure of the mean linear intercept (Lm) in Control and CS-exposed groups mice in a blinded manner, as previously described [46].

**Human tissue:** A portion of grossly normal lung tissue with size of approximately 1.0 cm2 in area and 0.5 cm of thickness was collected from the distal end of the lesion (≥5.0 cm) during the process of operation. The specimen was immediately snap frozen in liquid nitrogen (LN) for protein and RNA analysis. Lung tissues were embedded in paraffin or optimal cutting temperature (OCT) compound, and cut at a thickness of 4 μm for hematoxylin and eosin (HE). Tissue sections from all subjects were stained with HE for histopathologic examination. 5 randomly selected high magnification(400×) fields of each section were analysis.

**IHC assay**: briefly, the deparaffinized and rehydrated sections was heated antigen retrieval in 10 mM sodium citrate pH 6.0 for 15 minutes, then inactivated endogenous peroxidase with 0.3% hydrogen peroxide for 20 minutes and blocked with 5%BSA solution at 37℃ before incubating the primary antibody. The section incubated with anti-NOX4 antibody (1:100, Novus, Littleton, CO, USA), anti-α-SMA antibody (1:500, Abcam, Cambridge, UK), anti-TGF-β_1_(1:250, Abcam, Cambridge, UK) and anti-Collagen I antibody (1:200, Abcam, Cambridge, UK) overnight at 4℃. After washing three times, the section incubated with appropriate IgG antibody at 37℃ for 30min, then developed with DAB solution and hematoxylin counterstained. The images were captured with the Aperio-CS2 image capture device (Leica Biosystems, Germany) at 200x magnification.

**Preparation of cigarette smoke extract**

Cigarette smoke extract (CSE) was prepared as previously described[47]. CSE was made from the smoke of cigarette (Red Rose: 11 mg of tar and 10mg of nicotine), the mainstream smoke of two cigarette without filter was bubbled into a flask containing 10 ml of warm (37°C) phosphate-buﬀered saline (PBS) by use a vacuum-pump at a constant speed (each cigarette was smoked for 6 min), and filtered the solution through a 0.22μm pore filter (Millipore). To ensure standardization between batches of CSE, the absorbance was measured at 320nm on a spectrophotometer (Thermo, Scientific) and adjusted the optical density approximate at 1.0, then regarded as 100% CSE. 100% CSE was aliquoted and stored at -80℃, diluted with medium to applied to the cell at indicated concentration.

**Cell Culture**

Human bronchial smooth cells (HBSMCs) were purchased from ScienCell Research Laboratories (Cat.No.3400) (San Diego, CA, USA), cultured in complete Smooth Muscle Cell Medium (SMCM) containing 2% fetal bovine serum (FBS), smooth muscle cell growth supplement (SMCGS) and 1% penicillin/streptomycin solution (P/S) at 37℃ in a 5% CO_2_ incubator. The cells between passages 3-8 were used for subsequent experiments. After starvation for 24h in DMEM/F-12 medium with 0.5% FBS, the cells were pretreated with PD98059 (10μM), SB203580 (10μM), LY294002 (10μM) and N-acetylcysteine (1, 5 and10mM) for 1h before stimulation with TGF-β_1_.

**Western blot**

The protein expression of NOX4, α-SMA, TGF-β_1_ and Collagen I was determined by the western blotting. The total protein extracted from fresh mouse lung tissue and the harvested HBSMC in the RIPA lysis buffer (Thermo Scientific, Rockford, IL) with protease and phosphatase inhibitors.

**Protein extraction in lung tissue sample:** fresh lung tissue weighted and homogenized mechanically and enzymatically in a RPIA lysis buffer with protease and phosphatase inhibitors and lysed on ice for 15min, and then centrifuged at 14,000 g for 30min at 4℃, and transferred supernatants to pre-chill Ep tube before aliquoted at -80℃ before use. **Protein extraction in HBSMCs:** After various treatments, cells were washed with ice-cold PBS twice and lysed in a cold RIPA buffer containing protease inhibitor and phosphatase inhibitor cocktail for 20min on ice, then scraped the cells on ice and collected the lysates into the pre-chilled microcentrifuge tube. The lysates were centrifuged at 14,000 g for 30min at 4℃, and supernatants collected and stored at 80℃ before western blot analysis.

Total protein concentration was quantified by a BCA protein assay kit (Thermo Scientific). Equal amount of proteins was separated by SDS-PAGE, and transferred onto polyvinylidene difluoride membranes (pore size 0.2μm; Bio-Rad, Hercules, CA). The membranes were blocked with 5% non-fat milk (BD) at room temperature for 1 hour and incubated overnight at 4℃ with primary antibody. Primary antibody for NOX4 (1:500, Novus), α-SMA (1:1000, Abcam), Collagen I (1:2000, Abcam) and TGF-β_1_(1:2000, Abcam) were prepared in TBS with 5% non-fat milk and 0.1% Tween-20 (TBST). GAPDH (1:5000) was used as a loading control. The blots were incubated with horseradish peroxidase (HRP)-conjugated secondary antibody against either mouse or rabbit IgG for 1 hour at room temperature, and then developed using enhanced chemiluminescence (ECL) reagents (Thermo Fisher, USA). Immunoreactive signal was acquired by a Bio-Rad Chemidoc XRS/HQ. Densitometric analysis of all protein bands was performed using ImageJ Sorftware (NIH).

**Measurement of intracellular ROS**

Intracellular ROS levels were determined by measuring the mean fluorescence intensity of 2’-7’-dichlorodihydrofluorescein diacetate (H2DCFH-DA) (Cat. No. D399, Molecular Probes, USA). The mean fluorescence intensity of the cells was detected using the flow cytometer (BD Biosciences, San Jose, CA, USA) at 495-nm excitation and 525-nm emission. HBSMCs were cultured in completed SMCM for 24h in the 6-wells plates, then the cells stimulated with or without TGF-β_1_ (2ng/mL) for indicated time after pre-treatment. After treatment, the medium was removed and the cells were washed with warm PBS and incubated in serum-free DMEM/F-12 medium with 10μΜ H_2_DCFH-DA at 37℃ for 30min. At the end of incubation, cells were washed by warm PBS and then detached with trypsin/ED. The collections were centrifuged at 1200 rpm for 6 min, resuspended with PBS, the mean Fluorescence intensity of the cells was detected by the flow cytometer (BD Biosciences, San Jose, CA). The data was analyzed by the flowJo software.

**RNA isolation and quantitative real-time PCR**

Total RNA was extracted from cultured HBSMCs using TRIzol reagent (Invitrogen) and First-strand cDNA was synthesized from 500ng of total RNA using a PrimeScript RT reagent Kit (TaKaRa Biotechnology) according to the manufacturer’s protocols. The expression of *NOX4, α-SMA* and *GAPDH mRNA* was determined by quantitative real-time PCR reaction using SYBR Green PCR system (TaKaRa). The real time PCR reactions were performed for 40 cycles 95℃ for 10s, 60℃ for 30s in a 7500 Real Time PCR System (Applied Biosystems, Foster City, CA). The NOX4 and α-SMA primer sequences were obtained from a previous study[49], the internal reference gene was used as control to normalization. The relative mRNA amount in each sample was calculated using the 2^-△△Ct^ method, and. The data were normalized to GAPDH. The sequences of the primers used in qPCR were shown in Suppl. Table S2.

**Supp. Table S2. The sequences of the primers used for qPCR**

| GENE | The sequences of the primers |
| --- | --- |
| NOX4 | |
| forward | 5’-AGATGTTGGGGCTAGGATTG-3’ |
| reverse | 5’-TCTCCTGCTTGGAACCTTCT-3’ |
| α-SMA | |
| forward | 5’- GACCGAATGCAGAAGGAGAT-3’ |
| reverse | 5’-CCACCGATCCAGACAGAGTA-3’ |
| GAPDH | |
| forward | 5’-CAGCCTCAAGATCATCAGCA-3’ |
| reverse | 5’-ACAGTCTTCTGGGTGGCAGT-3’ |

**Immunofluorescence staining**

HBSMCs were plated on sterilized coverslips and grown in completed SMCM for 24h. After treatment, the cells were fixed with 4% paraformaldehyde for 15min at room temperature and subsequently incubated with 0.5%TritonX-100 in PBS for 10min. The cells were blocked with 5%BSA solution for 1h at 37℃ before incubated with primary antibody. The cells were incubated with rabbit anti-α-SMA (1:200, Abcam), rabbit anti-Collagen I (1:400, Abcam) overnight at 4℃. The cells were washed by 0.5%Tween-20 in PBS three times and incubated with goat anti-rabbit FITC-conjugated IgG (1:500, life) antibody for 1h at 37℃. Nuclei were stained with DAPI (1:500, sigma). Fluorescence was detected by Olympus fluorescence microscope.

Dual Immunofluorescence staining in HBSMC, the coverslips plated HBSMCs were fixed with 4% paraformaldehyde for 15min at room temperature and subsequently incubated with 0.5%TritonX-100 in PBS for 10min. The cells were blocked with 5%BSA solution for 1h at 37℃ before incubated with primary antibody. Primary antibody to rabbit anti-NOX4 (1:100, Novus) and mouse anti-α-SMA (1:200, Abcam) were co-incubated in PBS overnight at 4℃. After washed three times with 0.5%Tween-20 in PBS, the cells were incubated with goat anti-rabbit FITC-conjugated IgG (1:500, life) antibody and Cy3-conjugated goat anti-mouse (1:500) IgG antibody for 1h for 1h at 37℃. Nuclei were stained with DAPI (1:500, sigma). Fluorescence was detected by Olympus fluorescence microscope.

**Small interfering RNA (siRNA) transfection**

NOX4, SMAD3 and negative control (NC) siRNA were obtained from GenePharma (SuZhou, China). The sequence of siRNA as follow: NOX4 (sense: 5’- GAUCACAGCCUCUACAUAU-3’, antisense: 5’- AUAUGUAGAGGCUGUGAUC-3’), SMAD3(sense: 5’- UAGGCAGAAGCGCUCCGAA-3’, antisense: 5’- UUCGGAGCGCUUCUGCCUA-3’), NC (sense: 5’- UUCUCCGAACGUGUCACGU-3’, antisense: 5’- ACGUGACACGUUCGGAGAA-3’). HBSMCs were seed in six-well plates to 60% confluence, and transiently transfected with 50nM siRNA by 7.5μl Lipofectamine™ RNAmax (Thermo) for 6h, and then incubated with 2ng/mL TGF-β_1_ for indicated time to detected proliferation and the expression of protein and mRNA.

**Suppl. References**

1. He F, Liao B, Pu J, Li C, Zheng M, Huang L, Zhou Y, Zhao D, Li B, Ran P. Exposure to Ambient Particulate Matter Induced COPD in a Rat Model and a Description of the Underlying Mechanism. *Sci Rep* 2017; 7: 45666-45666.

2. Guan R, Wang J, Cai Z, Li Z, Wang L, Li Y, Xu J, Li D, Yao H, Liu W, Deng B, Lu W. Hydrogen sulfide attenuates cigarette smoke-induced airway remodeling by upregulating SIRT1 signaling pathway. *Redox Biol* 2020; 28: 101356-101356.

3. Liu X, Hao B, Ma A, He J, Liu X, Chen J. The Expression of NOX4 in Smooth Muscles of Small Airway Correlates with the Disease Severity of COPD. *Biomed Res Int* 2016; 2016: 2891810-2891810.

4. Hecker L, Vittal R, Jones T, Jagirdar R, Luckhardt TR, Horowitz JC, Pennathur S, Martinez FJ, Thannickal VJ. NADPH oxidase-4 mediates myofibroblast activation and fibrogenic responses to lung injury. *Nat Med* 2009; 15: 1077-1081.
